# Real-World Treatment Patterns and Survival Outcomes of Patients with Relapsed/Refractory Multiple Myeloma Treated with a Selinexor-Containing Triplet-Based Regimen

**DOI:** 10.3390/curroncol32050268

**Published:** 2025-05-02

**Authors:** Andrew Whiteley, Stephen C. Ijioma, David Ray, Spencer S. Langerman, Ellen Hu, Amy Pierre, Tomer Mark, Habte Yimer

**Affiliations:** 1 Texas Oncology-Baylor Sammons Cancer Center, 3410 Worth Street, Suite 400, Dallas, TX 75246, USA; 2 Karyopharm Therapeutics, Newton, MA 02459, USAdavid.ray@karyopharm.com (D.R.); tomer.mark@karyopharm.com (T.M.); 3 Flatiron Health, Inc., New York, NY 10013, USAdanyue.hu@flatiron.com (E.H.); amy.pierre@flatiron.com (A.P.); 4 Texas Oncology-Tyler, Tyler, TX 75702, USA; habte.yimer@usoncology.com

**Keywords:** real-world study, relapsed/refractory multiple myeloma, selinexor

## Abstract

Treatment for relapsed/refractory multiple myeloma (RRMM) is complex, with several classes of drugs that can be combined into doublet, triplet, or quadruplet regimens. Real-world studies can help to determine the optimal treatment sequences and dosing through observed usage of drugs outside of clinical trials. Previous clinical trials have demonstrated high rates of durable responses in the treatment of patients with triple-class-exposed RRMM with regimens containing selinexor, a first-in-class, orally available selective exportin 1 inhibitor. This study analyzed real-world treatment patterns and survival outcomes using a nationwide electronic health record-derived, deidentified database of patients with RRMM treated with an eligible selinexor-containing, triplet-based regimen, including combinations with dexamethasone and pomalidomide, bortezomib, carfilzomib, or daratumumab. Patients had a real-world overall survival (rwOS) of 14.7 months (95% CI: 10.6, 20.9) and a derived progression-free survival (dPFS) of 4.7 months (95% CI: 3.4, 6.7). Patients with previous exposure to anti-CD38 monoclonal antibodies (mAbs) in the most recent regimen prior to the selinexor treatment had numerically higher survival outcomes (rwOS, 20.9; dPFS, 8.7 months). These data suggest that, in the real-world setting, the use of selinexor triplet regimens is effective in patients with RRMM, especially those with prior exposure to an anti-CD38 mAb in the immediate prior line of therapy.

## 1. Introduction

Treatment for multiple myeloma (MM) has improved over the last few decades [1,2] with the introduction of new treatments and strategies, including proteasome inhibitors (PIs) (e.g., bortezomib, carfilzomib and ixazomib) [3,4,5,6], immunomodulatory therapies (IMiDs) (e.g., lenalidomide and pomalidomide) [7,8,9,10], and anti-CD38 monoclonal antibodies (mAbs) (e.g., daratumumab and isatuximab) [11,12,13,14], as well as combinations of these in doublet, triplet, or quadruplet regimens. However, even with these improvements, MM remains an incurable disease, with most patients eventually relapsing and/or developing refractory disease [15]. With the evolution of clinical practice patterns incorporating multiple classes of anti-myeloma agents in combination earlier in treatment, patients are more likely to become exposed or refractory to several drug classes after relatively few prior lines of therapy [16,17,18]. As patients live through the natural history of MM, multiple relapses leave increasingly more aggressive disease with fewer treatment options and are generally associated with poor prognosis [17,18].

Patients refractory to anti-CD38 mAbs in early lines of therapy are a particularly high-risk patient population with limited prospective clinical evidence supporting treatment recommendations. In the absence of randomized clinical trials, real-world studies become an important tool for evaluating optimal treatment strategies in difficult-to-treat populations and provide insights into treatment sequencing, dosing patterns, and effectiveness [19]. The MAMMOTH (Monoclonal Antibodies in Multiple Myeloma: Outcomes after Therapy Failure) study investigated the natural history and outcomes of patients with disease refractory to an anti-CD38 mAb [20]. The overall survival (OS) was 8.6 months, which indicates that patients who become refractory to an anti-CD38 mAb-based line of treatment and have progressed on multiple lines of treatment have poor outcomes [20]. Thus, the effectiveness of current treatment options is still limited in patients with heavily pretreated relapsed/refractory MM (RRMM), especially for those who have been exposed to an anti-CD38 mAb [18]. Other clinical trials with chimeric antigen receptor T cells and bispecific antibodies have shown activity in these patients; however, logistical burdens such as travel restrictions and hospitalization may put these therapies out of reach for patients with RRMM. In the case of bispecific antibodies, recommendations currently limit their use to after four prior lines of therapy.

There is a need for treatment options with different antigen targets and mechanisms of action that can improve outcomes in this patient population. The NCCN guidelines recommend that patients with RRMM should receive a new triplet regimen consisting of drugs or drug classes that they have not been exposed to within the last 6 months [1]. Immediate retreatment of daratumumab upon progression in MM is not recommended, with evidence suggesting the optimal time off therapy to be at least one year before rechallenging [21]. Preclinical and limited clinical evidence suggests that selinexor, a first-in-class, orally available selective exportin 1 (XPO1) inhibitor that has been approved by the FDA and recommended in guidelines in multiple combinations for the treatment of adult patients with RRMM, could be a potential treatment option in this setting [20,22,23]. In the current study, real-world treatment patterns and survival outcomes of patients with RRMM treated with a selinexor-containing, triplet-based regimen, particularly in those previously exposed to an anti-CD38 mAb in the immediate prior line of therapy, were examined.

## 2. Materials and Methods

### 2.1. Study Design and Patients

This is a real-world, longitudinal, retrospective cohort study that used secondary data of patients with a diagnosis of MM between 1 January 2011 and 31 January 2024 who received a selinexor-containing triplet regimen in the second line of therapy (2L) or above in the USA Flatiron Health Database. The primary objective of this study was to describe patient characteristics, treatment patterns and sequencing, and real-world clinical outcomes of patients with RRMM treated with a selinexor-containing, triplet-based regimen. This study leveraged a rules-based, oncologist-defined line of therapy (LOT) algorithm for MM that already existed in the Flatiron Health database.

### 2.2. Eligibility

Patients eligible for the study were aged ≥ 18 years at MM diagnosis and had evidence of eligible selinexor-containing triplet regimen treatment (XPd, XKd, XVd, and XDd) in ≥2L of at least two weeks’ duration. Patients were excluded from the study if there was evidence of selinexor utilization as a bridging therapy to chimeric antigen receptor T-cell therapy (CAR-T) or if treatment for MM started more than 30 days prior to the start of structured activity (e.g., laboratory values, prescribed drugs, etc.). The complete list of study inclusion and exclusion criteria is provided in Table 1.

### 2.3. Data Source

The source population was from the Flatiron Health Database, an electronic health record (EHR)-derived, deidentified database. At the time of this study, the database included 2800 cancer clinicians, with 1500 oncologists from more than 280 clinics and 800 unique sites of care in the United States. The Flatiron Health Database includes structured data in addition to unstructured data collected via technology-enabled data abstraction from physicians’ notes and other unstructured documents [24,25]. Approximately 75% of patients with data archived in the Flatiron Health Database received care from community cancer clinics, and the remaining 25% received care from academic clinics. In addition, the Flatiron Health Database offers derived data elements, including but not limited to LOT, derived-progression-free survival (dPFS), and mortality. Due to limitations in the capture of dosing details for oral therapies in structured drug administration data, the objectives of this study related to selinexor dosage using this data source were for exploratory purposes only.

### 2.4. Endpoints and Assessments

The primary objectives of this study were to describe patient characteristics, treatment patterns and sequencing, and dPFS and OS outcomes in real-world clinical settings. Derived PFS is a calculated measure of time during which a patient does not have disease progression based on data collected in a clinical trial or real-world setting [26,27]. Since data are less standardized in the latter, the calculations must consider data inconsistencies and missing information [28]. An algorithm-based approach to deriving disease progression based on reported monoclonal protein levels was used to derive progression in line with the International Myeloma Working Group criteria. Patients were followed retrospectively from an index date (start of the selinexor-containing triplet regimen) to the earliest occurrence of date of death, end of follow-up, or end of study period (i.e., the data cut-off date).

### 2.5. Statistical Analysis

Baseline demographic and clinical characteristics were collected for the study cohort. This included age at index LOT (continuous), age at index LOT (categorical: <65, 65–74, and ≥75 years of age), gender, geographic region, race, ethnicity, practice type, year of initial MM diagnosis, Eastern Cooperative Oncology Group (ECOG) performance score at index LOT, International Staging System (ISS) stage at initial diagnosis, cytogenetic risk status, individual cytogenetic abnormalities, immunoglobulin type, involved light chain type, stem cell transplant, time since initial diagnosis, year of index LOT, index LOT line number, index LOT regimen, and prior MM therapy. The Kaplan–Meier estimator was applied to estimate outcomes of interest for time-to-event analyses of dPFS and real-world OS (rwOS) from the initiation of the index LOT for the overall cohort and stratified subgroups. Kaplan–Meier curves were generated, along with tables displaying median survival times and 95% confidence intervals (CIs) for median survival. All time-to-event analyses yielded unadjusted estimates. A subgroup analysis on clinical outcomes was conducted in patients receiving anti-CD38 mAb treatment immediately prior to selinexor index LOT.

## 3. Results

### 3.1. Patients

**Figure 1:**
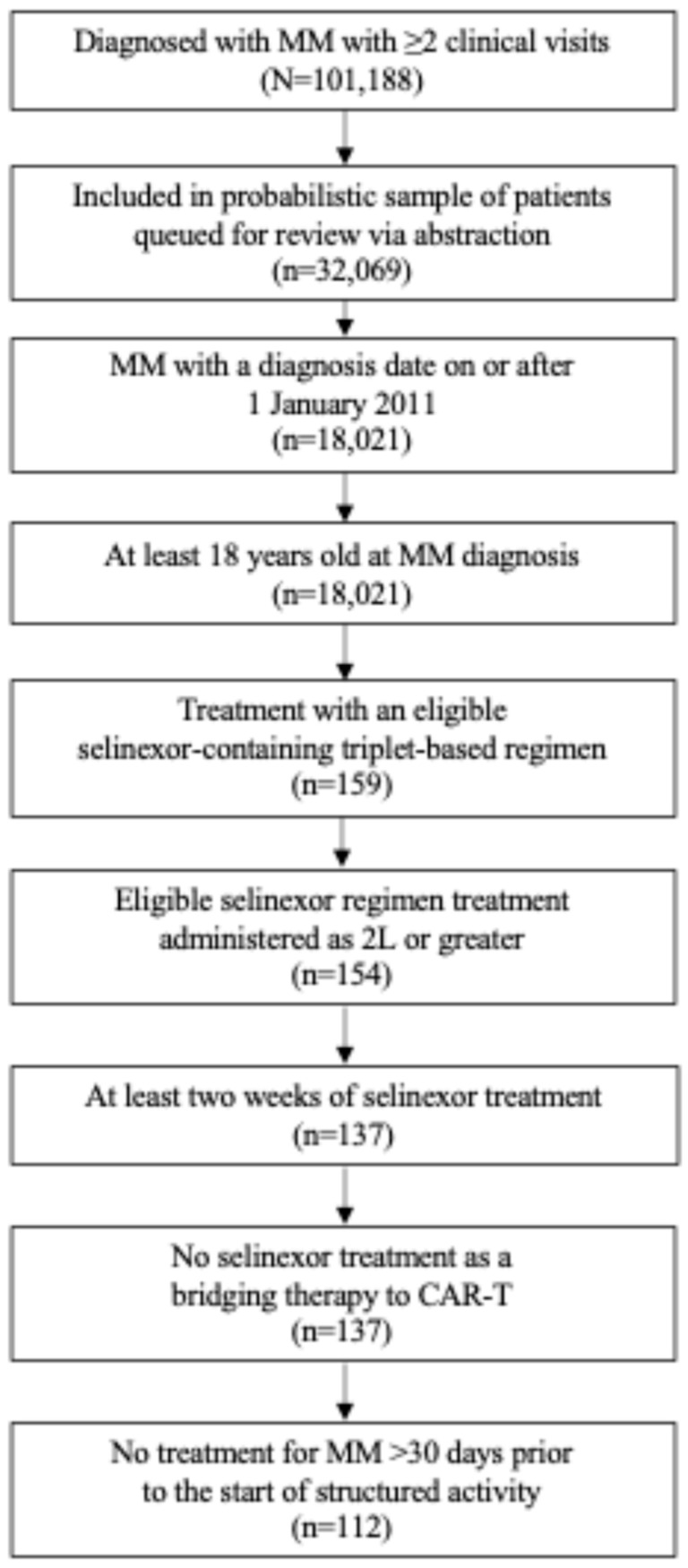
Study attrition. 2L, second line of therapy; MM, multiple myeloma.

Based on the cohort selection criteria, 112 eligible patients had initiated a selinexor triplet regimen (Figure 1). The median (interquartile range [IQR]) age of the patients at the index year was 69 (63, 76) years old and 54% were male. Overall, patients were predominantly White (75%), 92% identified as non-Hispanic or Latino, and 67% of the patients were seen in a community practice setting (Table 2).

Clinically, 55% of the patients had undergone an autologous hematopoietic stem cell transplant, and 29% were classified as having ISS stage III disease. The median (IQR) time from initial MM diagnosis to receiving the study index line was 58 (38, 80) months, and 94% received the index LOT at ≥4 lines of therapy. Prior to the study index LOT, most patients were triple-class exposed (95%), with previous exposure to lenalidomide (97%), bortezomib (96%), daratumumab (94%), pomalidomide (83%), and carfilzomib (77%). Baseline demographics and clinical characteristics among the patients receiving a selinexor triplet therapy immediately following an anti-CD38 mAb were similar to the overall cohort, with notable exceptions in LOT (index LOT at ≥4 lines, 94% vs. 84.2%) and the presence of high-risk cytogenetics (27% vs. 38%, Table 2).

### 3.2. Treatment Regimens

**Figure 2:**
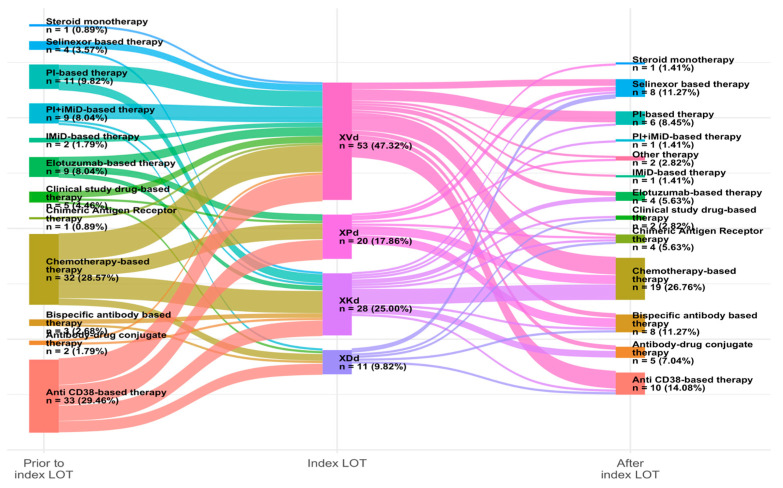
Distribution of therapies prior to and after index therapy. IMiD, immunomodulatory drug; PI, proteasome inhibitor; XDd, selinexor, daratumumab, and dexamethasone; XKd, selinexor, carfilzomib, and dexamethasone; XPd, selinexor, pomalidomide, and dexamethasone; XVd, selinexor, bortezomib, and dexamethasone.

Among the index line regimens, 47% of patients received XVd, 25% received XKd (selinexor, carfilzomib, and dexamethasone), 18% received XPd (selinexor, pomalidomide, and dexamethasone), and 9.8% received XDd (selinexor, daratumumab, and dexamethasone, Table 2). The most common therapies administered prior to the index line were anti-CD38-based and chemotherapy-based regimens, each accounting for approximately 29% of patients. Post-index line regimens included 27% of patients receiving chemotherapy and 14% receiving anti-CD38-based therapy. Notably, approximately 11% of patients continued with another selinexor-based therapy after the selinexor triplet regimen was completed. The full distribution of therapies prior to and post-index LOT is shown in Figure 2**.** There was a range of initial prescribed doses from 40 mg to 100 mg. Of these, 32% (24/77) of patients had an initial prescription of 100 mg selinexor, and 14 of these had modifications in the prescribed dose. In contrast, only one patient had a reduction in the prescribed dose among the 35% (27/77) of patients with an initial prescription of 60 mg. (Table 3).

### 3.3. Survival Outcomes in Real-World Clinical Settings

**Figure 3:**
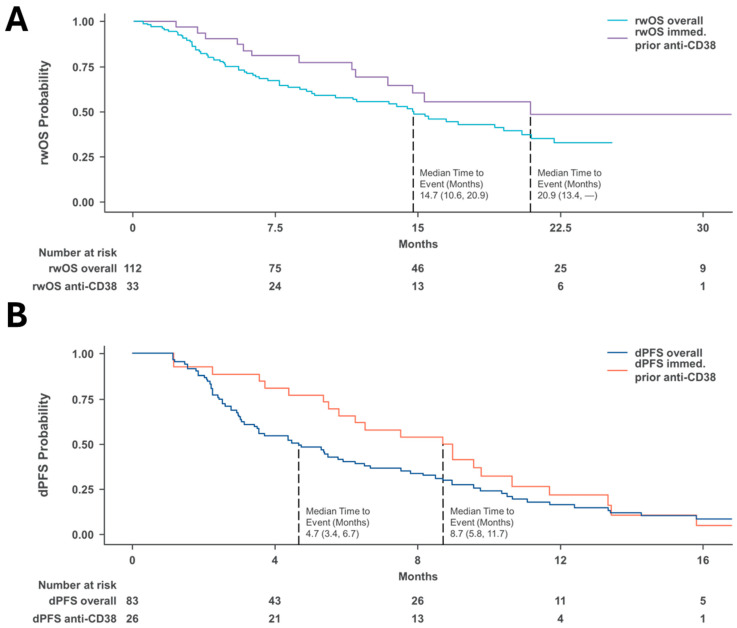
Survival. (**A**) Real-world overall survival. (**B**) Derived progression-free survival. CI, confidence interval; dPFS, derived progression-free survival; rwOS, real-world overall survival.

The median duration of follow-up for the study cohort was 9.4 months. The rwOS for the study cohort (*n* = 112) was 14.7 months ([95% CI: 10.6, 20.9], Figure 3A), while the overall dPFS for patients that were eligible for assessment (*n* = 83) was 4.7 months ([95% CI: 3.4, 6.7], Figure 3B). The subgroup of patients who received anti-CD38 mAb therapy immediately followed by the selinexor triplet index LOT had a numerically higher dPFS of 8.7 (95% CI: 5.8, 11.7) months compared to the overall cohort eligible for assessment, and the subgroup of patients who received anti-CD38 mAb therapy immediately prior to the selinexor triplet index LOT (*n* = 33) had a rwOS of 20.9 months ([95% CI: 13.4, NR], Table 4).

## 4. Discussion

Since the use of novel therapies and triplet combinations for MM, including PIs, IMiDs, and mAbs, has become more common, patients with heavily pretreated disease have fewer therapeutic options, which can result in poor outcomes and challenges for clinicians in determining the best way to proceed with treatment [19,24]. This real-world study investigated clinical outcomes of patients with heavily pretreated RRMM, including those who had been exposed to PIs, IMiDs, and anti-CD38 mAbs, and received selinexor treatment in 2L or greater. Currently, there is no standard of care for patients with RRMM in later lines of treatment. The Sankey diagram depicted in Figure 2 illustrates the many permutations possible and the lack of a defined sequencing pathway in the treatment of RRMM. The introduction of anti-CD38 mAbs, such as daratumumab and isatuximab, to the treatment of RRMM has shown impressive response rates in combination with IMiDs, PIs, or both, leading to use in the earlier lines and resulting in disease that is refractory to multiple agents upon relapse [25]. In this study, the patients were heavily pretreated, with 95% of patients having progressed after triple-class exposure and 94% receiving the selinexor triplet as the fourth or later line, thus leaving fewer options. The observed dPFS (4.7 months) and rwOS (14.7 months) outcomes among patients treated with XPd, XVd, XDd, and XKd suggest that selinexor triplet-based therapy is a reasonable choice among patients with RRMM, especially when viewed in light of the MAMMOTH study results [29].

Although a standard of care is not established in RRMM, there is a consensus that refractory status based on the last treatment is an important consideration when selecting the next therapy. The results among patients with immediate prior anti-CD38 mAb treatment represent a subgroup of candidates who could potentially benefit from selinexor-based treatment. The BOSTON and STOMP clinical trials report similar results among patients previously treated with an anti-CD38 mAb who received a selinexor-containing regimen (XPd, XVd or XKd); the median PFS was 10.2 months (95% CI: 7.3, 16.6) and median OS was 20.4 months (95% CI: 15.2, NE) [20]. The clinical results among this subgroup of patients are supported by preclinical studies with functional transcriptomics from the MM patient repository at the Moffitt Cancer Center, where gene clusters show an inverse relationship between daratumumab resistance and selinexor sensitivity [30].

Dosing migration from clinical development to real-world applications is a well-known phenomenon in clinical practice, particularly in MM with agents including, but not limited, to thalidomide, carfilzomib, and bortezomib. Selinexor was initially approved based on the STORM (NCT02336815) study, where patients with RRMM were administered 80 mg of selinexor plus dexamethasone twice weekly. Over the course of the study, 80% of patients had dose reductions, modifications, or changes in the dosing schedule to manage toxicities, including fatigue and nausea [31]. The subsequent BOSTON study evaluated selinexor in combination with bortezomib and dexamethasone at 100 mg weekly, yet dose modifications were common at a rate of 65%. This real-world study found that most patients were treated with an initial prescription of selinexor of less than 100 mg (69%) and that changes in prescribed dose were less common in patients with lower initially prescribed doses. This decrease in changes in prescribed amounts among patients with lower initial prescriptions provides a signal of potential improved tolerability and should be further studied to understand the impact on efficacy and effectiveness. The phase 3 study XPORT-MM-031 (NCT05028348; EMN29), evaluating selinexor at the 40 mg dose level in combination with pomalidomide and dexamethasone once-weekly immediately after anti-CD38 mAb therapy, may yield further insights into the impact of changed dosing on the efficacy and tolerability of selinexor.

Treatment patterns are not stagnant and require monitoring to understand how best to sequence and optimize treatments as new agents are developed. Multiple T-cell-redirecting therapies, such as CAR-T and bispecific antibodies, have recently been approved in RRMM, leading to questions on how best to sequence and combine these agents with the currently available treatments. These newer classes of immunological and cellular-based therapies present new questions on the importance of preserving and improving the interplay of the immune system, T-cell fitness, and tumor microenvironment, which is necessary for the optimal application of these agents. Preliminary preclinical and translational research suggests selinexor could ameliorate and potentially improve factors associated with T-cell exhaustion [32]. This study suggests that the administration of a selinexor-based regimen could improve patient survival after anti-CD38 mAb treatment. Sequencing of an anti-CD38-based regimen continued until disease progression, followed by a selinexor-based triplet and then followed by CAR-T or a bispecific antibody, may be a logical next step for study. Future research should focus on clinical outcomes associated with the implementation of selinexor combinations as a bridging therapy or holding therapy for CAR-T [33], as an interim therapy between bispecific antibodies, and other circumstances where T-cell health is a key factor in achieving successful outcomes.

### Limitations

We note several limitations to this study. First, the sample size was small due to the inclusion of patients who had previous exposure to selinexor, and the follow-up time was relatively short. Other limitations included those that are typically associated with real-world studies, such as the data source, immortal time bias, patients seeking care outside of the institution (leading to missing/incomplete data), patient loss to follow-up, possible misclassification of treatment patterns, generalized death dates, and missing information on MM progression. Future studies should include larger sample sizes with longer follow-up periods to validate the benefits of selinexor triplet therapies in the real-world setting. Dosage-related analyses were based on structured treatment data within the EHR. Selinexor is an oral drug with only the prescribed dose available; therefore, it is not possible to know the cumulative dose of selinexor to which a given patient was exposed after receiving a prescription. Future real-world analyses with chart abstraction to confirm the actual dose taken by the patient, or clinical studies, could be performed to attempt to verify these findings.

## 5. Conclusions

Multiple myeloma is a complex disease requiring treatment with multiple classes of drugs in combination. Patients will relapse, requiring different treatments and combinations to overcome drug resistance. This study suggests that the use of selinexor triplet regimens is effective in patients heavily pre-treated for RRMM, particularly in those patients with prior exposure to an anti-CD38 mAb in the immediate prior line of treatment in the real-world setting.

## Figures and Tables

**Table 1:** Patient eligibility criteria.

Inclusion Criteria	Exclusion Criteria
Included in Flatiron’s Multiple Myeloma (MM) Enhanced Data Mart (EDM) Diagnosed with MM (ICD-9 203.0x or ICD-10 C90.0x, C90) Has at least two documented clinical visits, on different days, occurring on or after 1 January 2011 Included in probabilistic sample of patients queued for review via abstraction ^1^ Has evidence of MM with a diagnosis date on or after 1 January 2011 At least 18 years old at MM diagnosis Has evidence of treatment with a selinexor-containing, triplet-based regimen (eligible selinexor regimen), according to Flatiron’s Line of Therapy business rules ^2^ Has evidence of eligible selinexor regimen treatment as any second line therapy (2L) or greater Has evidence of at least two weeks of selinexor treatment, defined as the presence of an abstracted oral span lasting more than 14 days	Lacking relevant unstructured documents in the Flatiron database for review by the abstraction team Has evidence of selinexor treatment as a bridging therapy to CAR-T, defined as treatment immediately prior to CAR-T cell infusion and lasting 30 days or fewer Has the start of treatment for MM, as captured through unstructured data, more than 30 days prior to the start of structured activity ^3^

2L, second line of therapy; CAR-T, chimeric antigen receptor T-cell therapy; EDM, Enhanced Data Mart; MM, multiple myeloma. ^1^ Patients were probabilistically sampled in order to ensure that an adequate number of patients were queued for chart review during the abstraction phase. A computer-based algorithm for probabilistic selection was used. ^2^ Eligible selinexor regimen defined as selinexor (received outside of a clinical trial) in combination with any of bortezomib, carfilzomib, daratumumab, or pomalidomide, and dexamethasone or prednisone. ^3^ Flatiron defines EHR structured activity as a recording of vital information, a medication administration, a non-canceled drug order, or a laboratory test/result report.

**Table 2:** Patient characteristics.

Characteristic, *n* (%)	Overall (*n* = 112)	Anti-CD38 mAb Treatment Immediately Prior to Selinexor-Triplet Regimen (*n* = 33)
**Age at index (year), median (IQR)**	69 (64, 76)	69 (68, 77)
Age, years *		
<65	38 (34)	8 (24)
65–74	41 (37)	10 (30)
≥75	33 (29)	15 (45)
**Starting weekly dosage, mg**		
100	24 (31)	6 (27) **
80	22 (29)	3 (14) **
60	27 (35)	11 (50) **
40	4 (5.2)	2 (9) **
**Gender**		
Male	61 (54)	17 (52)
Female	51 (46)	16 (48)
**Geographic Region**		
Midwest	11 (9.8)	4 (12)
Northeast	21 (19)	9 (27)
South	30 (27)	10 (30)
West	12 (11)	5 (15)
Unknown/Masked	38 (34)	5 (15)
**Race**		
Black or African American	15 (15)	3 (10)
Other Race	11 (11)	4 (13)
White	76 (75)	23 (77)
Unknown	0 (0)	0 (0)
**Ethnicity**		
Hispanic or Latino/Unknown	8 (8.4)	1 (3)
Not Hispanic or Latino	87 (92)	31 (97)
**Cytogenic risk**		
High	29 (26)	12 (38)
Standard	39 (35)	9 (28)
Unknown	39 (35)	11 (34)
**Index LOT line number**		
≤3L	7 (6.3)	5 (15)
4L	17 (15)	9 (27)
5L	31 (28)	14 (42)
6L	11 (9.8)	2 (6)
7L	18 (16)	1 (3)
≥8L	28 (25)	2 (6)
**Index LOT regimen**		
XVd	53 (47)	12 (36)
XKd	28 (25)	7 (21)
XDd	11 (9.8)	5 (15)
XPd	20 (18)	9 (27)
**ECOG performance status**		
0	30 (27)	9 (27)
1	50 (45)	16 (48)
≥2	17 (15)	4 (12)
Unknown	15 (13)	4 (12)
**ISS stage**		
I	22 (20)	6 (18)
II	34 (30)	7 (21)
III	32 (29)	10 (30)
Unknown/not documented	30 (27)	10 (30)
**Previous therapy exposures**		
Bortezomib	107 (96)	28 (85)
Carfilzomib	86 (77)	23 (70)
Ixazomib	31 (28)	8 (24)
Daratumumab	105 (94)	32 (97)
Isatuximab	5 (4.5)	2 (6)
Elotuzumab	27 (24)	6 (18)
Lenalidomide	109 (97)	32 (97)
Pomalidomide	93 (83)	24 (73)
Thalidomide	5 (4.5)	0 (0)
**Autologous Hematopoietic Stem Cell Transplant**		
Prior transplant	62 (55)	16 (48)
No prior transplant	50 (45)	17 (52)

L, number of line of therapy; ECOG, Eastern Cooperative Oncology Group; IQR, interquartile range; ISS, International Staging System; LOT, line of therapy; XDd, selinexor, daratumumab, and dexamethasone; XKd, selinexor, carfilzomib, and dexamethasone; XPd, selinexor, pomalidomide, and dexamethasone; XVd, selinexor, bortezomib, and dexamethasone. * Patients with a birth year of [Data Cutoff Year - 85] or earlier may have an adjusted birth year in Flatiron Health datasets due to patient deidentification requirements. ** Based on patients with available data (*n* = 22).

**Table 3:** Summary of dosing information.

	Overall (*n* = 77)	100 mg (*n* = 24)	80 mg (*n* = 22)	60 mg (*n* = 27)	40 mg (*n* = 4)
**Had change in prescribed dose, *n*, (%)**	22 (29)	14 (58)	7 (32)	1 (3.7)	0 (0)
**Selinexor Regimen *n*, (%)**					
XVd	39 (51)	20 (83)	12 (55)	6 (22)	1 (25)
XKd	18 (23)	1 (4.2)	8 (36)	8 (30)	1 (25)
XDd	7 (9.1)	3 (13)	1 (4.5)	2 (7.4)	1 (25)
XPd	13 (17)	0 (0)	1 (4.5)	11 (41)	1 (25)

XDd, selinexor, daratumumab, and dexamethasone; XKd, selinexor, carfilzomib, and dexamethasone; XPd, selinexor, pomalidomide, and dexamethasone; XVd, selinexor, bortezomib, and dexamethasone. Results from the cohort reporting dosing patterns are part of an exploratory analysis.

**Table 4:** Median derived progression-free survival and real-world overall survival from start date of the index line of therapy.

Characteristic	dPFS Months (95% CI)	rwOS Months (95% CI)
**Overall**	4.7 (3.4, 6.7)	14.7 (10.6, 20.9)
**Post-anti-CD38**	8.7 (5.8, 11.7)	20.9 (13.4, NE)

CI, confidence interval; dPFS, derived progression-free survival; NE, not evaluable; rwOS, real-world overall survival.

## Data Availability

The datasets generated and/or analyzed during the current study are available from the corresponding author upon reasonable request.
